# Dynamic cerebral autoregulation in infants undergoing major non-cardiac surgery

**DOI:** 10.1177/0271678X251406519

**Published:** 2026-01-18

**Authors:** Sigrid D. Vik, Hans Torp, Anders H. Jarmund, Turid Follestad, Ragnhild Støen, Siri Ann Nyrnes

**Affiliations:** 1 Children’s Clinic, St. Olavs University Hospital, Trondheim University Hospital, Trondheim, Norway; 2 Department of Circulation and Medical Imaging, Norwegian University of Science and Technology (NTNU), Trondheim, Norway; 3 Department of Public Health and Nursing, Norwegian University of Science and Technology (NTNU), Trondheim, Norway; 4 Department of Clinical and Molecular Medicine, Norwegian University of Science and Technology (NTNU), Trondheim, Norway

**Keywords:** Brain injury, cerebral hemodynamics, dynamic cerebral autoregulation, infant, surgery

## Abstract

Dynamic cerebral autoregulation (CAR) is the capacity in the cerebral vessels to attenuate the effect of rapid changes in blood pressure (BP). NeoDoppler, a novel ultrasound system, monitors cerebral blood flow velocity (CBFV) through an open fontanel continuously. Our primary aim was to evaluate dynamic CAR using continuous Doppler in infants undergoing major non-cardiac surgery. The pressure-flow relationship was evaluated in the time- and frequency domain, and the two methods were compared. The secondary aim was to compare this with near-infrared spectroscopy (NIRS). CBFV and regional oxygenation were synchronized with invasive BP monitoring. Nineteen infants (ten preterm) were included. In the time-domain, NeoDoppler showed that the mean proportion of time with impaired CAR was highest during surgery (0.59, 95% CI 0.48–0.70) compared with anesthesia before start of surgery (0.41, 95% CI 0.31–0.52, *p* = 0.040), and recovery (0.25 95% CI 0.13-0.36, *p* < 0.001). There was no evidence of different time profiles when using NeoDoppler in the time- versus the frequency domain (*p* = 0.165), or between NeoDoppler and NIRS in the time-domain (*p* = 0.167). In contrast to NIRS, NeoDoppler enabled direct evaluation of the cerebrovascular reactivity. Continuous Doppler monitoring combined with invasive BP monitoring enables a unique possibility to evaluate the pressure-flow relationship.

## Introduction

Major non-cardiac surgery in infants is associated with brain injuries and neurodevelopmental delay.^1–4^ Factors such as surgical stress, the exposure to anesthetic drugs and disturbances in cerebral blood flow (CBF) may contribute to these brain injuries in the immature brain.^5–7^ Cerebral autoregulation (CAR) is one of the mechanisms involved to ensure stable and sufficient perfusion of the brain despite changes in cerebral perfusion pressure. In a clinical setting, systemic blood pressure (BP) is the closest surrogate for cerebral perfusion pressure. Within certain BP limits, the cerebral vessels have the capacity to change their vascular tone, thereby preventing cerebral hypo- and hyperperfusion.^8,9^ The optimal BP for maintaining intact CAR is however unclear.^5,9,10^ The autoregulatory capacity can be divided into static and dynamic CAR. Static CAR refers to the ability to maintain stable CBF as a response to gradual changes in BP over time, whereas dynamic CAR is the capacity to attenuate rapid changes in BP that occur within seconds and minutes.^8^ Such rapid changes in BP can occur during surgery and in combination with impaired dynamic CAR this increases the risk of brain injuries.^8^ Knowledge of the autoregulatory capacity in infants is, however, not fully understood since an optimal method to evaluate the pressure-flow relationship is lacking. This hampers the possibility to prevent brain injuries and adverse neurological outcomes in preterm infants and sick neonates, including anesthetized infants.

The assessment of dynamic CAR in infants has been challenging both due to the lack of an optimal real-time method with sufficient high temporal resolution to monitor CBF and due to the difficulties in quantifying the relationship between BP and CBF. Near infrared spectroscopy (NIRS) has been the most used surrogate for CBF in the evaluation of CAR in infants.^11^ NIRS measure regional cerebral oxygenation (rcSO2) in the superficial part of the frontal cortex and may indirectly reflect changes in CBF under several assumptions.^12,13^ Despite extensive research, knowledge of dynamic CAR in infants is still scarce and the use of NIRS has not shown clinical benefit.^14,15^ Cerebral Doppler provides direct information on cerebral hemodynamics by visualizing the Doppler waveform within each heartbeat.^16^ The use of Doppler to evaluate CAR in infants has previously been limited by the lack of the possibility to apply continuous monitoring. NeoDoppler is a novel ultrasound system which enables continuous monitoring of cerebral blood flow velocity (CBFV) in infants with an open fontanel.^17,18^ In combination with continuous BP monitoring this enables a unique possibility to evaluate the pressure-flow relationship in infants.^19^

Our primary aim was to evaluate dynamic CAR and cerebrovascular reactivity using continuous Doppler in infants undergoing major non-cardiac surgery. The pressure-flow relationship was evaluated in the time- and frequency domain, and the two methods were compared. The secondary aims were to compare this with NIRS and explore the association between CAR capacity and BP.

## Materials and methods

### Study design

In this prospective observational study, infants undergoing major non-cardiac surgery at the Department of pediatric surgery, St. Olavs University Hospital, Trondheim, Norway, were included between May 2020 and May 2022. Background variables were collected from the infant’s medical records and included surgical diagnosis, gestational age, postnatal age at surgery, birth weight and weight at surgery.

**Figure 1.**
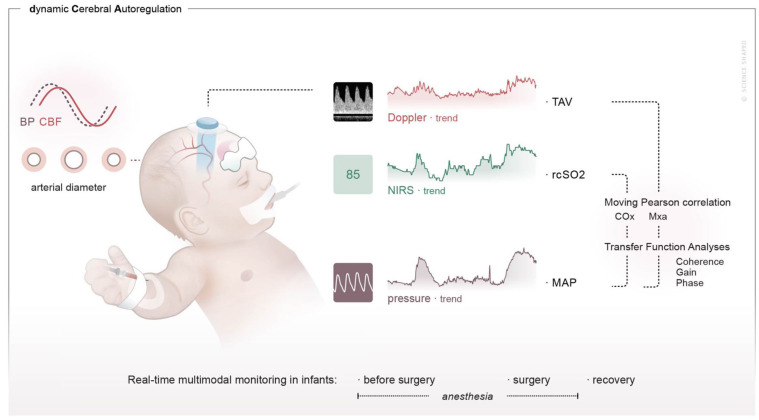
Illustration of the monitoring set up and study design. The figure shows cerebral circulation monitoring with NeoDoppler and NIRS, which are separately synchronized with continuous blood pressure monitoring during the three main periods: anesthesia before start of surgery, during surgery and during recovery. Two different methods were used to quantify the relationship between blood pressure and cerebral blood flow: the moving Pearson correlation and transfer function analysis. BP: blood pressure; CBF: cerebral blood flow; NIRS: near-infrared spectroscopy; TAV: time averaged velocity; rcSO2: regional cerebral oxygenation; MAP: mean arterial pressure; COx: cerebral oxygenation index (the moving Pearson correlation of rcS02 and MAP); Mxa: mean flow index (the moving Pearson correlation of TAV and MAP). Coherence, gain and phase are the output variables of transfer function analyses.

An arterial line for invasive BP monitoring was inserted as per clinical practice after induction of anesthesia, and BP was continuously monitored during anesthesia, surgery and recovery. As surrogate markers of cerebral perfusion, CBFV was continuously assessed using NeoDoppler, and regional cerebral oxygenation (rcSO2) was continuously assessed using NIRS. The readings from both modalities were blinded to the clinical team. The NeoDoppler probe was placed over the infant’s anterior fontanel before induction of anesthesia and provided continuous information on CBFV parameters: peak systolic velocity (PSV), time-averaged velocity (TAV) and end-diastolic velocity (EDV). The calculated Doppler indices reflecting vascular resistance were pulsatility index (PSV-EDV/TAV) and resistive index (PSV-EDV/PSV). The Doppler parameters and their relationship to cerebral hemodynamics are as follows; PSV is primarily influenced by systemic factors such as cardiac output and blood pressure, TAV is more directly related to cerebral perfusion, with increased TAV potentially reflecting elevated blood pressure or reduced peripheral resistance. End-diastolic velocity is considered the most sensitive marker to changes in cerebral hemodynamics, such as changes in peripheral resistance and/or diastolic blood pressure.^16^ A NIRS sensor for infants and neonates, INVOS^TM^ 5100c OxyAlertTm (Medtronic Parkway, Minneapolis, MN, USA) was placed on the infant’s forehead before induction of anesthesia and measured continuous rcSO2. A NIRS reading of 95 is the maximum value measured with the INVOS system and may reflect high oxygen supply or arterial oxygenation. A NIRS reading of 95 without variations does not reflect changes in CBF and cannot be used in autoregulation analyses. The monitoring set-up is shown in Figure 1.

Sevoflurane anesthesia combined with intermittent administration of fentanyl and rocuronium was the standard anesthetic management. Hemodynamic management was at the discretion of the responsible anesthesiologist and consisted of fluid administration and/or vasopressor. Details of anesthetic and hemodynamic management as well as events during monitoring were registered at the bedside by the first author who was present during all surgeries.

### Data capture and post-processing

Blood pressure, heart rate, arterial oxygen saturation, Doppler velocity parameters and rcSO2 were recorded and synchronized in real-time from start of monitoring until end of recovery. Real-time data acquisition was done with an in-house, custom-made software developed in MATLAB (MathWorks®R2021a). Synchronizing of the monitoring variables was done using pyMIND, an open-source Python-based software designed to acquire and integrate multi-modal scientific data from medical devices. The software was modified in-house and captured data from the Philips IntelliVue, such as BP, heart rate and arterial oxygen saturation, and the system was extended to capture NIRS from the stand alone INVOS monitor. On the receiver side, a common time axis was maintained to which all measurements were synchronized. The highest available temporal resolution was captured for all signals, which was 424 Hz for NeoDoppler, 0.2 Hz for NIRS and 130 Hz for BP, and saved into HDF format for offline processing. Artefacts were manually removed prior to the autoregulation analyses through visual inspection of the trend curves. Outliers in BP, Doppler, and NIRS were identified and excluded from the dataset. This ensured that only high-quality, synchronized data segments were included in the analyses. The concentration of expired sevoflurane, the fraction of inspired oxygen (FiO2), end-tidal partial pressure of carbon oxide and rectal temperature were recorded every 30 s (s) from start of anesthesia until end of surgery. During recovery, FiO2 was recorded every 30 s, and capillary pCO2 was measured once.

### Evaluation of dynamic cerebral autoregulation

**Figure 2.**
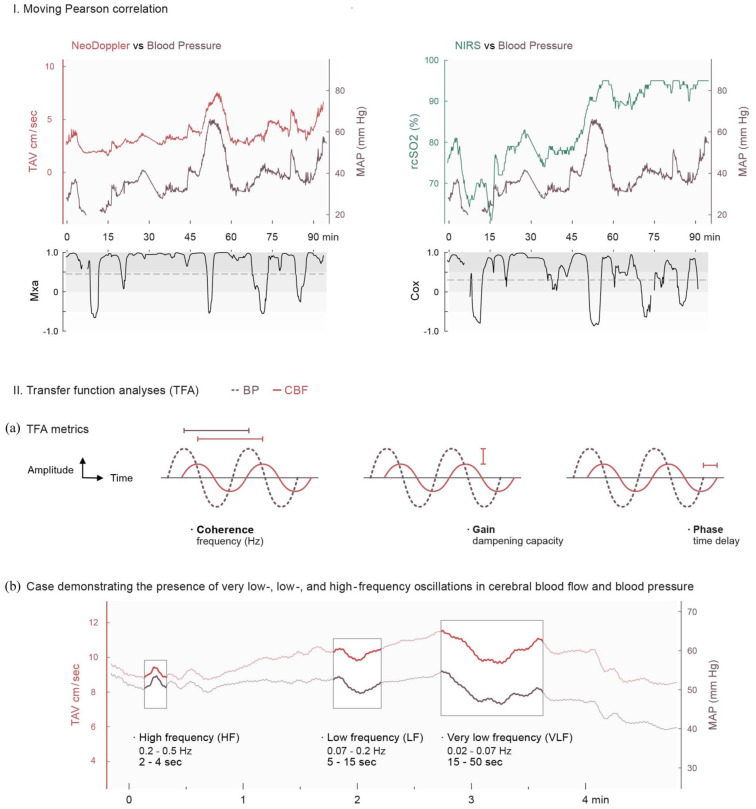
The moving Pearson correlation (I) and Transfer Function Analyses (II). (2_I) The relationship between cerebral blood flow and blood pressure evaluated with Moving Pearson correlation in one infant with congenital diaphragmatic hernia during surgery. Upper panel shows synchronized data of cerebral blood flow (NeoDoppler left (TAV) and NIRS right (rcSO2) and blood pressure (MAP). The lower panel shows the corresponding results of the mean flow index (left), and the cerebral oxygenation index, COx (right), with higher values indicating impaired cerebral autoregulation. The dotted line indicates the threshold for impaired CAR (0.45 for Mxa and 0.3 for COx). In this case, the mean proportion of time with impaired CAR measured with Mxa is 0.78, and measured with COx is 0.6. (2_II): Explanation of transfer function analysis, (a) explanation of the output measures coherence, gain and phase of transfer function analysis. (b) synchronized data of TAV and MAP during four minutes in one patient with M. Hirschsprung during surgery. Oscillations with the same frequency are present in both blood pressure and cerebral blood flow and is not attenuated. This gives values of coherence and gain as follows: Coherence VLF 0.74, LF 0.87 and HF 0.79 and gain VLF 1.3, gain LF and HF both 0.99. BP: blood pressure; CBF: cerebral blood flow; TAV: time averaged velocity; MAP: mean flow index; sec: seconds; min: minutes.

Dynamic CAR was assessed in the time-domain by using the moving Pearson correlation coefficient, and in the frequency domain by using transfer function analysis (TFA). These are two commonly used methods for the evaluation of dynamic CAR in the neonatal population.^9,11,20^ Custom-made MATLAB scripts (by HT and AHJ) were developed for both methods. Invasively measured mean arterial pressure (MAP) was used as a surrogate for cerebral perfusion pressure. Mean arterial pressure was automatically calculated as the time-averaged pressure over each cardiac cycle from the arterial pressure waveform, sampled at 130 Hz. As surrogates for CBF, we used TAV and rcSO2 (Figures 1 and 2). Time-averaged velocity was derived from continuous Doppler measurements obtained with NeoDoppler and automatically calculated as the TAV over each cardiac cycle from the maximum velocity curve (TAVmax) with a sampling frequency of 424 Hz.

### Time-domain analysis (Figure 2_I)

By using the moving Pearson correlation coefficient, the mean flow index (Mxa) was calculated as the linear relationship between MAP and TAV, while the cerebral oxygenation index (COx) represents the linear relationship between MAP and rcSO2. Originally, Mx was calculated as the correlation between cerebral perfusion pressure and TAV.^21^ When cerebral perfusion pressure is replaced with invasively measured MAP, the index is referred to as Mxa.^21^ To calculate Mxa, we employed a moving window approach. Each window was 300 s long and comprised 30 paired data points from 10-second averages of MAP and TAV. Mxa was calculated for each heartbeat using a moving window approach, resulting in overlapping data segments where individual data points contributed to multiple calculations. This approach enabled tracking temporal trends in Mxa values throughout the monitoring period. The moving window was applied continuously from the insertion of the arterial line (after induction of anesthesia), during surgery and until end of recovery, generating a continuous stream of Mxa values. Correlation values ranged from minus one to plus one, with higher values indicating reduced autoregulatory capacity.^22^ The same approach was used to calculate COx. Based on the published literature, thresholds of Mxa above 0.45 and COx above 0.3 were used to define impaired CAR.^23–25^ We then calculated the percentage of time during which Mxa and COx exceeded their respective thresholds, thereby quantifying the duration of impaired CAR across the different monitoring periods.

### Frequency-domain analyses (Figure 2_II)

The transfer function analysis (TFA) evaluates the relationship between CBF and BP in the frequency domain, where oscillations with different frequencies are identified. The in-house, custom-made MATLAB script used for TFA in this study was developed based on the recommendations from Claassen et al.^26^ and Panerai et al.^27^ The frequency range of interest includes the very low frequencies (VLF; 0.02–0.07 Hz), the low frequencies (LF; 0.07–0.2 Hz) and the high frequencies (HF; 0.2–0.5 Hz). The frequencies were identified within a moving 300-second window with 50% overlap of the windows. The calculated TFA metrics are coherence, gain and phase (Figure 2_IIa). Coherence ranges from zero to plus one, where higher values indicate that oscillations with the same frequency are present in both CBF and BP, suggesting impaired CAR.^26^ A coherence value above 0.3 was set as the threshold for impaired CAR.^11,27,28^ Persistently low coherence across all frequency bands indicates poor data quality and the results are considered not reliable.^26^ Gain indicates the dampening capacity of the cerebral vessels, that is, how much of the amplitude of the oscillation is attenuated. We calculated gain by normalizing both CBF and BP, that is, as percentage change from baseline for both CBF and BP.^27^ Gain equal to one indicates total loss of CAR if coherence is high, gain below one indicates that some of the signal is attenuated. Gain above one indicates a relative higher response in CBF than in BP, that is, which may indicate impaired CAR or the presence of oscillations in CBF which are not generated from BP. Phase describes the time-lag between the signals, with a low phase indicating impaired CAR.^27^ For Doppler TFA, we present the TFA metrics coherence and gain for all the three frequencies. For NIRS TFA, with a sampling frequency of 0.2 Hz, the VLF and LF oscillations were analyzed.

### Statistical analyses

#### Defining periods

Statistical analyses were performed using IBM SPSS Statistic version 29.0. Three main periods were defined for dynamic CAR analyses; P1) ‘Before surgery’; after induction of anesthesia, from insertion of arterial line until start of surgery, representing anesthesia without exposure to surgery, P2) ‘Surgery’; from start of surgery until end of surgery and P3) ‘Recovery’; the period during recovery with synchronized data of BP and CBF.

#### Evaluation of dynamic cerebral autoregulation over time

**Figure 3.**
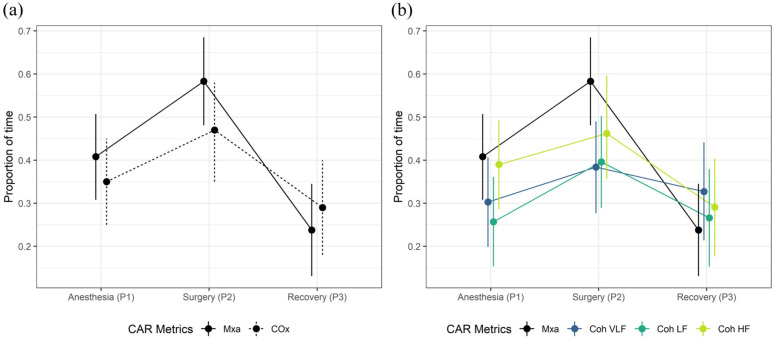
The mean proportion of time with impaired cerebral autoregulation during anesthesia before start of surgery (P1), during surgery (P2) and during recovery (P3), including 95% confidence interval. (a) Mean flow index (Mxa) versus cerebral oxygenation index (COx). (b) Mean flow index (Mxa) versus Doppler coherence. Mxa: mean flow index; COx: cerebral oxygenation index; TFA: transfer function analysis; Coh: coherence; VLF: very low frequency; LF: low frequency; HF: high frequency.

Linear mixed models (LMM) were used to investigate how CAR changes over time and to compare the methods Mxa, COx, and Doppler coherence. The LMM was also used to investigate how gain changes over time during the period P1, P2 and P3. The use of LMM enables inclusion of infants with missing data at some time points. The mean proportion of time, calculated as the percentage of time with values above the threshold indicating impaired CAR (Mxa = 0.45, COx = 0.3 and Coherence = 0.3), was used as dependent variable, and method (Mxa, COx or Doppler coherence) and time point (P1, P2 and P3) were included as categorical independent variables. The model includes the interaction between method and time. The distribution of the residuals for Mxa, Cox and Doppler coherence was explored with QQ plots and tests of normality and found to be normally distributed (Shapiro-Wilk, *p* = 0.48). The distribution of the residuals for gain was not normally distributed and gain was therefore log-transformed. The residuals of the log transformed data were normally distributed, explored with histogram, QQ plots and tests of normality (Shapiro-Wilk, *p* = 0.32). Estimated means and mean difference for the proportion of time with impaired CAR are presented with corresponding 95% confidence interval (CI) and *p*-values (Table 2 and Figure 3).

#### Cerebral autoregulation and blood pressure

Hypotension was defined as MAP <35 mmHg,^29^ and we used an independent samples t-test to see if hypotension was associated with reduced CAR capacity. To investigate the presence of an autoregulatory plateau in the infants, the average over 1 min for TAV was plotted against the average over 1 min for MAP in a scatter plot, with MAP ‘bins’ of 2 mmHg on the *x*-axis.^23^

For all analyses, *p*-values below 0.05 were considered to indicate statistically significant results. Bonferroni correction was applied to p-values for multiple pairwise comparisons for the method and time variables in the LMMs, to preserve a 5% significance level for the corresponding overall tests.

### Visual inspection of cerebrovascular reactivity

The NeoDoppler trend curves and the corresponding BP were visually inspected to detect if changes in CBFV directly can reveal information related to the cerebrovascular reactivity.

### Safety

During monitoring with NeoDoppler, values of mechanical index (MI) and thermal index (TI) were continuously visible. The highest transmitted energy is located at the skin surface, decreasing with increasing depth. The skin was inspected after removing the probe. The clinical team was free to remove the probe if the monitoring set up was interfering with the clinical management of the infants.

The study was conducted in accordance with the Declaration of Helsinki and approved by the Regional Ethics Committee for Medical and Health Research Ethics in Central Norway, REC Central (registration number 2017/314) for studies involving humans. The study was also approved by the Norwegian Directorate of Health (Reference: 17/15181-11), and The Norwegian Medicines Agency (Reference 19/05458). Informed, written parental consent was obtained before surgery by a research nurse or the first author.

## Results

### Patient data

During the study period, a total of 19 infants had invasive BP monitoring, providing data eligible for CAR analysis inclusion. Demographic data for these infants are presented in Table 1. Surgical groups included esophageal, duodenal and anal atresia (7), abdominal wall defects (4), M. Hirschsprung (2), Congenital diaphragmatic hernia (2), patent ductus arteriosus (2) and other major surgery (2). Respiratory, anesthetic and hemodynamic management are presented in Supplemental Table 2 (Table S2). All infants were mechanically ventilated during all periods of monitoring. Vasopressor was used in five infants during anesthesia and surgery, and in four infants during recovery. Median time from induction of anesthesia to insertion of an arterial line was 23 minutes (min), range 7–49 min. The measurements of CBFV, NIRS, BP, heart rate, arterial oxygen saturation and temperature during monitoring are presented in Supplemental Table S1.

**Table 1.** Demographic data.

	*N* (%)	Median (range min-max)
Total	19 (100)	
Sex (male)	12 (63.2)	
Gestational age (weeks)		36.6 (24.6–41.4)
Preterm (<37 weeks)	10 (52.6)	
Term (⩾37 weeks)	9 (47.4)	
Birth weight (gram)		2280 (591–5595)
<2500	10 (52.6)	
⩾2500	9 (47.4)	
Postmenstrual age (weeks) at surgery		37.1 (28.6–60)
Age at surgery (days)		2.08 (0.3–140)
>7 days	5 (26.3)	
< 7 days	14 (73.7)	
Weight at surgery (gram)		2280 (930–7755)
Surgery Groups		
Esophageal, duodenal and anal atresia	7 (36.8)	
Gastroschisis and omphalocele	4 (21.1)	
M. Hirschsprung	2 (10.5)	
Congenital diaphragmatic hernia	2 (10.5)	
Patent ductus arteriosus	2 (10.5)	
Ascites, percutaneous gastric tube	1 (5.3)	
Ileostomy reversal	1 (5.3)	

### Dynamic cerebral autoregulation

Using NeoDoppler, the median time with CAR analyses during P1 was 56 min (range 26–132 min), during P2 180.5 min (range 31–331 min) and during P3 106 min (range 15–202 min). For NIRS, median time with CAR analyses during P1 was 55.5 min (range 26–132 min), during P2 184 min (range 75–331 min) and during P3 106 min (15–202 min). Supplemental Table S6 shows an overview of infants with incomplete data across the monitoring periods (P1–P3) and the reasons for data incompleteness.

#### Mxa versus Doppler coherence

The estimated mean proportion of time with impaired autoregulation for Mxa was highest during P2 (0.59, 95% CI 0.48–0.70), compared with P1 (0.41, 95% CI 0.31–0.52, *p* = 0.040, Bonferroni corrected *p*-value) and P3 (0.25, 95% CI 0.13–0.36, *p* < 0.001, Bonferroni corrected *p*-value), based on an LMM with method-by-time interaction including Mxa and Doppler Coherence VFL, LF and HF (Table 2). Doppler Coherence VLF, LF and HF had a smaller estimated mean reduction from P2 to P3 compared with Mxa (Table 2 and Figure 3). However, the method-by-time interaction was not statistically significant (*p* = 0.165), such that there was no evidence of different time profiles between the methods (Table 2 and Figure 3). Since the interaction effect was not significant, the estimated mean difference between Mxa and Doppler VLF, LF and HF was calculated from an LMM without the interaction effect. The estimated mean Mxa was consequently higher than the means for Doppler Coherence, with the largest mean difference between Mxa and Doppler Coherence LF (0.12, 95% CI 0.03–0.20, *p* = 0.038 (Bonferroni corrected *p*-value), Table 2 and Figure 3).

**Table 2.** The estimated mean proportion of time with impaired autoregulation for Mxa, Cox and NeoDoppler coherence VLF, LF and HF, and the estimated mean difference between the methods.

	With the method-by-time interaction							Without the method-by-time interaction		
		Anesthesia (P1)		Surgery (P2)		Recovery (P3)				
	*p*-values	Estimated means (95% CI)	*p*-values*	Estimated means (95% CI)	*p*-values*	Estimated means (95% CI)	*p*-values *	Mean difference (estimated)	95% CI (of mean difference)	*p*-values*
Mxa versus Coherence										
Method-by-time (overall)	0.165									
Mxa		0.41 (0.31 to 0.52)	0.074^a^	0.59 (0.48 to 0.70)	0.040^b^	0.25 (0.13 to 0.36)	<0.001^c^			
NeoDoppler coherence VLF		0.30 (0.20 to 0.41)	0.100^a^	0.38 (0.28 to 0.49)	0.766^b^	0.33 (0.21 to 0.44)	0.100^c^			
NeoDoppler coherence LF		0.26 (0.15 to 0.36)	0.100^a^	0.40 (0.29 to 0.50)	0.156^b^	0.27 (0.15 to 0.38)	0.255^c^			
NeoDoppler coherence HF		0.39 (0.29 to 0.50)	0.548^a^	0.46 (0.36 to 0.57)	0.922^b^	0.29 (0.18 to 0.40)	0.070^c^			
Method (overall)										0.033
Mxa versus Coh VLF								0.09	0.00 to 0.17	0.255
Mxa versus Coh LF								0.12	0.03 to 0.20	0.038
Mxa versus Coh HF								0.04	−0.04 to 0.12	1.0
Time (overall)										<0.001
Surgery (P2) versus anesthesia (P1)								0.12	0.05 to 0.19	0.004
Recovery (P3) versus anesthesia (P1)								−0.06	−0.13 to 0.02	0.386
Recovery (P3) versus surgery (P2)								−0.18	−0.25 to −0.10	<0.001
Mxa versus Cox										
Method-by-time (overall)	0.167									
Mxa		0.41 (0.31 to 0.51)	0.023^a^	0.58 (0.48 to 0.69)	0.011^b^	0.24 (0.13 to 0.36)	<0.001^c^			
Cox		0.35 (0.25 to 0.45)	0.959^a^	0.47 (0.35 to 0.58)	0.230^b^	0.29 (0.18 to 0.40)	0.030^c^			
Mxa versus COx								0.04	−0.03 to 0.11	0.246
Time (overall)										<0.001
Surgery (P2) versus anesthesia (P1)								0.15	0.06 to 0.24	0.003
Recovery (P3) versus anesthesia (P1)								−0.12	−0.21 to −0.03	0.037
Recovery (P3) versus surgery (P2)								−0.27	−0.36 to −0.12	<0.001

Mxa: mean flow index; COx: cerebral oxygenation index; Coh: coherence; VLF: very low frequency; LF: low frequency; HF: high frequency; CI: confidence interval. The estimated mean difference and the corresponding 95% confidence interval are calculated from a linear mixed model without the method-by-time interaction.

a P1 versus P3, ^b^P2 versus P1, ^c^P2 versus P3.

* Bonferroni corrected *p*-values for pairwise comparisons

#### Mxa versus Cox

The estimated mean proportion of time with impaired CAR for COx in the LMM with the method-by-time interaction including Mxa are shown in Table 2. There was no evidence of different time profiles between Mxa and COx (*p* = 0.167 for interaction, Table 2 and Figure 3). The mean difference between Mxa and COx in the model without interaction was 0.04 (95% CI −0.03 to 0.11, *p* = 0.246).

#### Doppler gain

Doppler gain VLF, LF and HF were higher during P3 than during P2 (*p* < 0.001, Supplemental, Table S3).

#### NIRS coherence

NIRS coherence VLF and LF was low during all the three periods of monitoring and the results were considered not reliable (Supplemental Table S4).

### Cerebral autoregulation and blood pressure

During anesthesia before start of surgery, infants with MAP below 35 mmHg (N = 6) had a higher mean proportion of time with impaired CAR (Mxa 0.55; SD 0.16, Cox 0.57; SD 0.21) than infants with MAP above 35 mmHg (N = 11, Mxa 0.35; SD 0.21, Cox 0.26, SD 0.18, *p* = 0.02 for both Mxa and COx). During surgery, only one infant had MAP below 35 mmHg. We were not able to display a clear autoregulatory plateau in any patients in our data. Supplemental Figure S1 shows the autoregulatory curves from five infants during all the three periods of monitoring, P1–P3 (Supplemental Figure S1). Supplemental Table S5 presents the corresponding CAR metrics Mxa and COx for these five infants.

### Visual inspection of cerebrovascular reactivity

**Figure 4.**
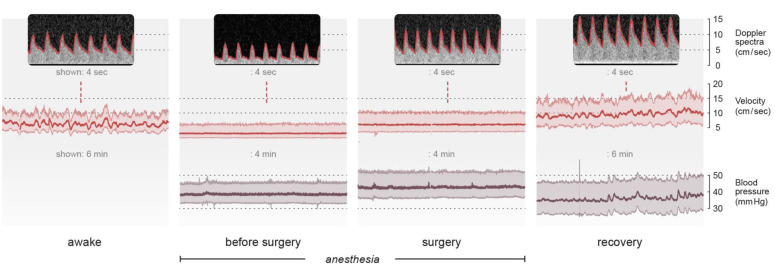
Variations in the presence of oscillations in cerebral blood flow velocity and the corresponding blood pressure in one infant with omphalocele from awake state until recovery. During anesthesia before start of surgery and during surgery no oscillations are observed in either velocity or blood pressure. During surgery blood flow velocity and blood pressure increase compared with during anesthesia before start of surgery. During recovery, very low and low frequency oscillations are presented in velocity without corresponding oscillations in blood pressure. In the same infant, before induction of anesthesia, very low- and low frequency oscillations are observed. Sec = seconds, min = minutes.

Figure 4 shows a representative example of absent and present oscillations in the VLF and LF range in CBFV during the different periods (P1–P3), with the corresponding BP. During recovery, the observed oscillations in CBFV are not present in BP. During anesthesia before start of surgery, and during surgery, no oscillations are observed in CBFV or in the corresponding BP (Figure 4). In the infant represented in Figure 4, oscillations in CBFV similar to what was seen during recovery, were also observed before induction of anesthesia. The mean proportion of time with impaired CAR estimated with Mxa in this patient was 0.49 during P1 and 0.46 during P2, whereas during recovery CAR was preserved all the time.

**Figure 5.**
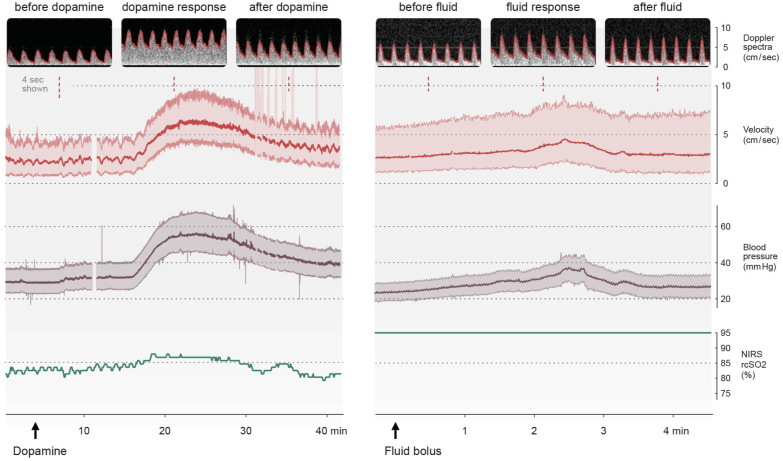
The effect of vasopressor and fluid bolus on cerebral blood flow velocity and blood pressure. Figure 5 (left panel): an increase in blood pressure and velocity as a response to start of dopamine infusion (5 mcg/kg/min). Compared to baseline, the increase in peak systolic pressure was 78%, mean arterial pressure was 76% and end diastolic pressure was 77%. Compared with baseline, the increase in peak systolic velocity was 68%, time averaged velocity was 104 % and in end diastolic velocity was 191%. Resistive index decreases from 0.77 from start of dopamine to 0.52 at the blood pressure top. The absolute value of rcSO2 increases from 81% to 87% from start of dopamine to the blood pressure top. Mxa and COx indicate impaired CAR at the blood pressure top with correlations values of 0.88 and 0.64, respectively. Figure 5 (right panel) shows the response in BP and CBFV on manually given fluid bolus, Plasma-Lyte 10 mL/kg over two minutes. The measured value of NIRS is 95%, without variations, and does not reflect changes in CBF.

Figure 5 (left panel) shows a representative example of the effect of dopamine on CBFV with a direct observed response in BP and CBFV. Compared to baseline, the increase in peak systolic pressure was 78%, in MAP was 76% and in end diastolic pressure was 77%. Compared to baseline, the increase in PSV was 68%, in TAV was 104 % and in EDV was 191%. The resistive index decreases from 0.77 from start of dopamine to 0.52 at the top of the blood pressure wave. The measurements of rcSO2 varied from 81% to 87% (Figure 5, left panel). Mxa and COx indicate impaired CAR with a correlation of 0.88 and 0.64, respectively, at the top of the blood pressure wave. Figure 5 (right panel) shows a representative example of the response to fluid bolus, with a corresponding increase in both BP and CBFV. NIRS measures 95% without variations and does not reflect changes in CBF.

### Safety

During all periods of monitoring, MI and TI at the skin surface were consistently 0.06 and 0.57, respectively. There were no visible skin marks after several hours of monitoring. None of the infants needed the probe to be removed during monitoring.

## Discussion

This is the first study to use continuous Doppler monitoring to evaluate dynamic CAR in infants undergoing major non-cardiac surgery. Our findings show that the proportion of time with impaired CAR was highest during surgery, compared to anesthesia prior to incision and recovery. There was no evidence of different time profiles of impaired dynamic CAR between Mxa, COx and Doppler coherence. The VLF and LF oscillations observed in CBFV may directly reflect cerebrovascular reactivity, potentially indicating a normal physiological response to maintain stable cerebral perfusion. These oscillations were not observed with NIRS.

The highest proportion of time with impaired dynamic CAR in our study was during surgery, assessed with NeoDoppler in both the time- and frequency domain, and with NIRS in the time-domain. Sevoflurane reduces cerebral metabolism and acts directly as a vasodilator on cerebral vessels.^29–31^ The combination of sevoflurane and inflammation triggered by surgical stress may have an additive negative impact on CAR.^5^ The autoregulatory capacity in our patients improved after end of surgery and sevoflurane anesthesia and was then comparable to what has been reported in other sick neonates without general anesthesia and surgical interventions.^32,33^ Comparable studies evaluating dynamic CAR during non-cardiac surgery in infants are scarce.^9^ Kuik et al evaluated CAR by using NIRS in 19 infants before, during and after surgery and found impaired CAR in 63% of the infants during surgery.^34^ In accordance with our findings, all infants in that study had preserved CAR during recovery. Another study found high correlation between rcSO2 and MAP in 15 children less than 1 year of age receiving sevoflurane during craniofacial surgery indicating poor dynamic CAR.^35^ The effect of dopamine on CAR is controversial^36,37^ and we had only five infants with vasopressor during surgery. However, in all five of these infants there was an immediate response in CBFV when vasopressor was initiated. As demonstrated in Figure 5, the increase in EDV and TAV was higher than in end diastolic blood pressure and MAP, and the calculated resistive index decreases. Since EDV is considered the most sensitive marker for changes in cerebral hemodynamics the enormous response in EDV may reflect the combination of increased BP (due to increased cardiac output) and a decrease in peripheral resistance (due to sevoflurane), and may reflect impaired CAR as the vessels are not able to attenuate the increase in BP. Future studies are needed to evaluate this relationship and the response in the cerebral vessels. Hypercapnia increases the risk of pressure-passive circulation, however the end-tidal partial pressure of carbon oxide was within normal range in all patients in our study.

To quantify impaired CAR, we calculated the percentage of time during which Mxa, COx and Doppler coherence exceeded predefined thresholds. Instead of evaluating CAR at isolated time points, this allowed us to estimate the burden of impaired CAR across different monitoring periods and enables a more comprehensive assessment of dynamic CAR. We found no evidence of different time profiles for the proportion of time with impaired CAR assessed with Mxa, COx and Doppler coherence. However, although not statistically significant, Mxa consequently measured a higher mean proportion of time with impaired CAR than COx during anesthesia before start of surgery and during surgery, with the highest mean difference during surgery. This may indicate that COx tends to underestimate the proportion of impaired CAR during the periods where the CAR capacity is most affected. Evaluating dynamic CAR is challenging, and there is no universally accepted gold-standard method.^38^ Both time- and frequency domain methods are subject to methodological variability and a lack of standardization.^22,26,39^ Correlation based methods are considered more straightforward than TFA, whereas TFA may better evaluate the cerebrovascular reactivity involved in dynamic CAR.^22,27,40,41^ However, it remains uncertain whether CAR can be accurately quantified or meaningfully dichotomized into categories such as intact or impaired.^11, 42^ The NIRS-based moving Pearson correlation coefficient, calculated as COx, is a frequently used method to assess CAR in the neonatal population.^11,43^ Its Doppler-based counterpart, Mxa, is less commonly used in neonates, as continuous Doppler monitoring until recently has not been suitable in this population. In adult studies, Mxa has demonstrated poor validity and reliability,^21,44^ raising concerns about its appropriateness as a time-domain measure of CAR.^45^ However, in our study, Mxa, COx and Doppler coherence all indicated that dynamic CAR was most affected during surgery compared to anesthesia and recovery. Given the physiological plausibility that autoregulatory capacity is most vulnerable during surgery, our findings may support that the methods used in our study are measuring the same underlying physiological response involved in the regulation of CBF.

The directly observed changes in the vascular tone, that is, the VLF and LF oscillations, observed with NeoDoppler were not observed with NIRS. These VLF and LF oscillations in CBFV have also been reported in healthy term born neonates monitored with NeoDoppler.^17,46^ In adults, the presence of oscillations with the same characteristics is related to increased cerebrovascular reactivity and considered neuroprotective.^46^ Since the oscillations were not observed in BP, we speculate that these oscillations are caused by the dynamic change in the vascular tone of the cerebral vessels and may indicate preserved cerebrovascular reactivity. The Doppler gain for the VLF and LF oscillations was significantly higher during recovery than during surgery. This may indicate reduced presence of oscillations during surgery. In combination with the increased proportion of time with impaired CAR during surgery this supports that these oscillations may play an important role in the cerebrovascular reactivity. Further studies are needed to investigate if these oscillations may indicate preserved CAR and their clinical relevance considering brain injuries and long-term outcome.

We were not able to identify an autoregulatory plateau in any patients in our data. However, since we evaluate dynamic CAR, that is, the effect of rapid changes in BP on CBF, we may not expect to find a classic autoregulatory plateau. We do, however, expect that dynamic CAR also has an optimal BP range which is associated with optimal dampening capacity of the cerebral vessels. General recommendations on BP management may, however, not be easy as individual differences exist and the CAR capacity is influenced by the clinical condition of the infants and clinical interventions.^9,10,32,47,48^ Although BP monitoring is the most used single parameter to guide hemodynamic stability and to indirectly ensure stable and sufficient cerebral perfusion^10, 49–52^ we do not know the lower limit of BP. The lack of knowledge considering optimal BP hampers the possibility to prevent brain injuries in sick newborn infants, including anesthetized infants. Individualized hemodynamic assessment to guide intraoperative hemodynamic management is therefore highly needed.^49,53,54^ Continuous cerebral circulation monitoring may have the potential to more accurately identify the optimal BP range to keep the cerebral circulation stable.

### Strengths and limitations

One limitation of this study is the relatively small sample size. This study was an exploratory pilot study, and the number of 19 infants was based on the availability of eligible patients with invasive BP monitoring during the study period. It was not ethically appropriate to design a study that required the placement of an arterial line solely for research purposes in this vulnerable population. The relatively small number of infants included was partly compensated for by the large amount of synchronized data of BP and CBF with high temporal resolution, optimal for the evaluation of dynamic CAR.

The NIRS-based TFA in our study was questionable since NIRS coherence was low across both the VLF and LF ranges. This may be due to the sampling frequency of the INVOS^TM^ 5100c device, which is 0.2 Hz. According to the Nyquist theory, accurate signal analysis requires a sampling frequency at least twice the highest frequency of interest. Consequently, our setup limits reliable analysis to frequencies up to 0.1 Hz, thereby excluding part of the LF range (defined as 0.07–0.2 Hz). Additionally, the nature of the NIRS measurements may have been influenced by perioperative physiological conditions. Specifically, the high fraction of inspired oxygen and reduced cerebral metabolism during anesthesia and surgery may have contributed to the in general high and stable NIRS values. This may have affected the fluctuations within the frequency range of interest.

A methodological limitation of this study are the cut-off values for the CAR metrics Mxa, COx, and Coherence. Although these thresholds are based in previous literature, they remain inherently arbitrary and may influence the interpretation of cerebral autoregulation status.

During monitoring, the infants were relatively stable hemodynamically and they all underwent similar anesthetic management. The first author was present throughout the entire monitoring period.

Artefact removal in this study was performed manually through visual inspection of the trend curves. For future clinical applications, automating this process would enhance consistency and reproducibility.

### Future perspectives

Synchronized data of continuous Doppler and BP with high temporal resolution enables a unique possibility to evaluate the pressure-flow relationship within each cardiac cycle in infants. This may provide important insight into aspects such as the critical closure pressure of the cerebral vessels, cerebral perfusion pressure and peripheral resistance. Further investigation of these aspects is necessary and may contribute to the development of improved guidelines for hemodynamic management. If cerebral monitoring with NeoDoppler can be used to guide hemodynamic management this may contribute to prevent brain injuries.

## Conclusion

Continuous Doppler monitoring revealed that the mean proportion of time with impaired dynamic CAR was highest during surgery compared to anesthesia and recovery in infants undergoing major non-cardiac surgery. There was no evidence of different time profiles between Mxa, COx and Doppler coherence. In contrast to NIRS, continuous Doppler monitoring can reveal valuable information on the cerebrovascular reactivity involved in dynamic CAR. This may contribute to new insight into the pressure-flow relationship in this vulnerable patient group.

## Supplemental Material

sj-docx-1-jcb-10.1177_0271678X251406519 – Supplemental material for Dynamic cerebral autoregulation in infants undergoing major non-cardiac surgery Supplemental material, sj-docx-1-jcb-10.1177_0271678X251406519 for Dynamic cerebral autoregulation in infants undergoing major non-cardiac surgery by Sigrid D. Vik, Hans Torp, Anders H. Jarmund, Turid Follestad, Ragnhild Støen and Siri Ann Nyrnes in Journal of Cerebral Blood Flow & Metabolism
