# Supplementary material for: Dynamic cerebral autoregulation in infants undergoing major non-cardiac surgery
Source: J Cereb Blood Flow Metab. 2026 Jan 18:0271678X251406519. Online ahead of print. doi: 10.1177/0271678X251406519 (PMC12815638; doi:10.1177/0271678X251406519)
Supplement: sj-docx-1-jcb-10.1177_0271678X251406519 – Supplemental material for Dynamic cerebral autoregulation in infants undergoing major non-cardiac surgery [file sj-docx-1-jcb-10.1177_0271678X251406519.docx]

**Supplementary materials**

**Table S1:** Measurements of cerebral blood flow velocities, pulsatility and resistive index, near-infrared spectroscopy and vital parameters

**Table S2:** Respiratory, anesthetic and hemodynamic management during anesthesia, surgery and recovery

**Table S3:** The estimated means of Doppler gain during anesthesia, surgery and recovery

**Table S4:** NIRS coherence during the three periods of monitoring (median, range minimum-maximum)

**Figure S1:** Autoregulatory curves from five infants during anesthesia (before start surgery, P1), surgery (P2) and recovery (P3)

**Table S5:** Individual infant data for mean flow index (Mxa) and cerebral oxygenation index (COx) for five infants with autoregulatory curves presented in Figure S1

**Table S6:** Infants with incomplete data across the monitoring periods and the reasons for data incompleteness.

Table S1: Measurements of cerebral blood flow velocities, pulsatility and resistive index, near-infrared spectroscopy and vital parameters

|  |  |  |  |
| --- | --- | --- | --- |
|  | **ANESTHESIA** | |  |
|  | **Before surgery (P1)** | **During surgery (P2)** | **During recovery (P3)** |
|  | N=17* | N=16** | N=14*** |
| PSV median (IQR) | 7.97 (6.17-12.73) | 8.72 (6.91-15.27) | 11.15 (7.85-12.88) |
| TAV median (IQR) | 4.39 (3.01-5.62) | 4.63 (3.72-7.2) | 6.68 (4.95-8.14) |
| EDV median (IQR) | 1.64 (1.35-2.46) | 2.15 (1.91-2.90) | 3.18 (2.76-4.47) |
| PI median (IQR) | 1.63 (1.39-2.05) | 1.37 (1.27-1.63) | 1.01 (0.88-1.39) |
| RI median (IQR) | 0.79 (0.72-0.85) | 0.73 (0.7-0.79) | 0.63 (0.59-0.73) |
| NIRS median (IQR) | 79.00 (71.98-90.70) | 84.66 (70.51-91.77) | 79.95 (72.36-86.92) |
| PSP median (IQR) | 47.52 (43.34-55.62) | 52.98 (50.04-57.00) | 62.05 (50.38-71.01) |
| MAP median (IQR) | 38.57 (32.46-42.20) | 41.52 (37.49-46.09) | 50.72 (43.40-64.37) |
| EDP median (IQR) | 30.65 (24.02-35.31) | 34.44 (30.20-38.06) | 44.32 (34.53-54.58) |
| HR median (IQR) | 127.70 (118.17-143.65) | 137.35 (125.42-152.79) | 132.03 (117.83-152.02) |
| Sp02 median (IQR) | 97.10 (95.80-97.66) | 97.50 (96.40-98.85) | 95.34 (93.55-97.03) |
| Temperature rectal median (IQR) | 36.39 (35.39-37.10) | 36.92 (36.27-37.16) | 37.05 (36.38-37.10)^a^ |
| PSV = peak systolic velocity, TAV = time average velocity, EDV = end-diastolic velocity, PI = pulsatility index, RI = resistive index, | | | |
| NIRS = Near-infrared spectroscopy, PSP = peak systolic pressure, MAP = mean arterial pressure, EDP = end diastolic pressure, | | | |
| HR = heart rate, Sp02= oxygen saturation, IQR = interquartile range, *Two infants missing P1 and P2, ** Two infants missing P1 and P2, | | | |
| and one infant missing P2 and P3, ***One infant missing P2 and P3, four infants missing P3, ^a^N=5 | | |  |

Table S2: Respiratory, anesthetic and hemodynamic management during anesthesia, surgery and recovery

|  | **N** | **Median**  **(range min-max)** |
| --- | --- | --- |
| **Respiratory management** |  |  |
| Inspired oxygen before start of surgery (%) | 16 | 49.19 (30.78-72.80) |
| Inspired oxygen during surgery (%) | 15 | 48.80 (30.00-59.17) |
| Inspired oxygen during recovery (%) | 14 | 24.86 (20.99-34.73) |
| End tidal C02 before start of surgery | 14 | 4.27 (3.7-6.63) |
| End tidal CO2 during surgery | 13 | 4.36 (3.77-6.21) |
| PcO2 capillary during recovery | 13 | 5.8 (4.32-7.39) |
| **Anesthetic management** |  |  |
| Sevoflurane* before start of surgery (%) | 14 | 1.48 (1.03-2.46) |
| Sevoflurane* during surgery (%) | 13 | 1.58 (0.9-2.82) |
| Fentanyl before start of surgery (µg/kg) | 17 | 2.14 (0-4.30) |
| Fentanyl during surgery (µg/kg) | 16 | 5.96 (0.64-31.11) |
| Rocuronium before start of surgery (µg/kg) | 17 | 0.52 (0-4.3) |
| Rocuronium during surgery (µg/kg) | 16 | 1.43 (0-4.3) |
| **Sedation during recovery** |  |  |
| None | 6 |  |
| Fentanyl infusion during recovery (µg/kg/h) | 4 | 1.5 (1.0-2.0) |
| Fentanyl bolus during recovery (µg/kg) | 1 | 1 |
| Morphine infusion during recovery (µg/kg/h) | 1 | 20.0 |
| Morphine bolus during recovery (µg/kg) | 2 | 0.08 (0.05-0.1) |
| **Hemodynamic management** |  |  |
| Vasopressor |  | **(Range min-max)** |
| Dopamine during surgery (µg/kg/min) | 4 | 2.05-10 |
| Dopamine during recovery (µg/kg/min) | 3 | 3.0-10 |
| Adrenalin during surgery (µg/kg/min) | 1 | 0.08-0.12 |
| Adrenalin during recovery (µg/kg/min) | 1 | 0.11 |
| *Expired sevoflurane concentration |  |  |

Table S3: The estimated means of Doppler gain during anesthesia, surgery and recovery

|  | **Anesthesia (P1)** | | **Surgery (P2)** | | **Recovery (P3)** | |
| --- | --- | --- | --- | --- | --- | --- |
| **Gain** | Estimated means (95% CI) | p-values* | Estimated means (95% CI) | p-values* | Estimated means (95% CI) | p-values* |
| Doppler gain VLF | 1.96 (1.38-2.54) | <0.001^a^ | 1.850 (1.268-2.431) | 1.000^b^ | 3.167 (2.557-3.778) | <0.001^c^ |
| Doppler gain LF | 2.56 (1.99-3.13) | 0.118^a^ | 1.883 (1.301-2.464) | 0.216^b^ | 3.262 (2.652-3.873) | <0.001^c^ |
| Doppler gain HF | 2.25 (1.69-2.82) | <0.001^a^ | 1.860 (1.279-2.442) | 0.504^b^ | 3.683 (3.083-4.304) | <0.001^c^ |
| Results from linear mixed model, p-values from log transformed gain data since the residuals were not normally distributed, *Bonferroni corrected p-values for pairwise comparisons. | | | | | |  |
| ^a^P1vsP3, ^b^P2vsP1, ^c^P2vsP3, VLF=very low frequency, LF= low frequency, HF= high frequency, CI= confidence interval | | | | |  |  |

Table S4: NIRS coherence during the three periods of monitoring (median, range minimum-maximum)

|  |  |  |  |  |  |  |
| --- | --- | --- | --- | --- | --- | --- |
|  | **Before (P1)** | | **During (P2)** | | **After (P3)** | |
| **Coherence** | **VLF** | **LF** | **VLF** | **LF** | **VLF** | **LF** |
| Median | 0,15 | 0,16 | 0,17 | 0,15 | 0,16 | 0,15 |
| Minimum | 0,07 | 0,08 | 0,11 | 0,11 | 0,13 | 0,11 |
| Maximum | 0,22 | 0,24 | 0,22 | 0,17 | 0,36 | 0,20 |
| P1 = anesthesia, before start of surgery, P2 = surgery, P3 = recovery, VLF = very low frequency, LF = low frequency | | | | | | |

Figure S1: Autoregulatory curves from five infants during anesthesia (before start surgery, P1), surgery (P2) and recovery (P3)

*
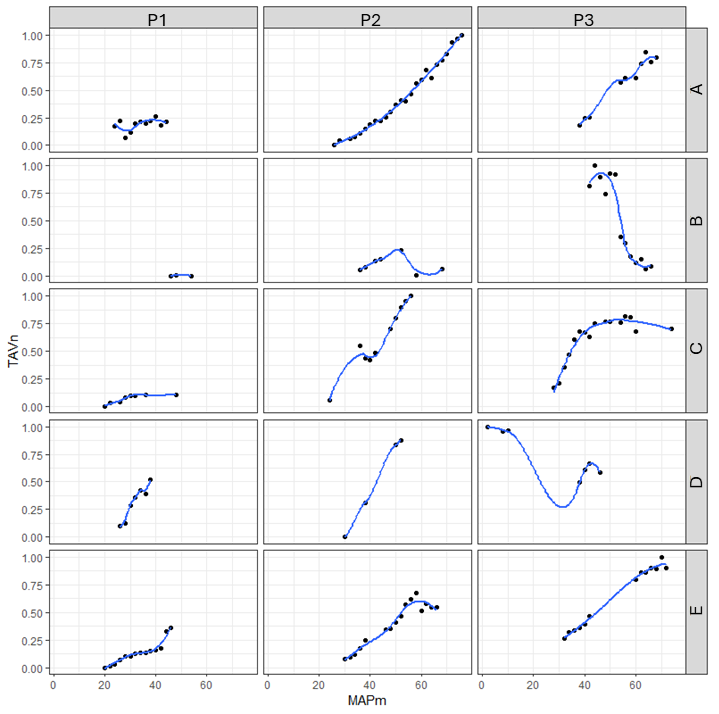
*

The figure shows autoregulatory curves from five infants (A-E) during the three monitoring periods (P1 to P3). Based on Mxa and COx two infants (B and C) demonstrated low mean percentage of time with impaired CAR, while three infants (A, D and E) demonstrated a high mean percentage of time with impaired autoregulation (A, D, E). The mean percentage of time with impaired CAR for Mxa and COx for these five infants are presented in Table S5 related to this Figure (see below).

Table S5: Individual infant data for mean flow index (Mxa) and cerebral oxygenation index (COx) for five infants with autoregulatory curves presented in Figure S1

| **Patients** | **Methods** | **P1** | **P2** | **P3** |
| --- | --- | --- | --- | --- |
| **A Adominal wall defect Preterm** | Mxa | 65 % | 76 % | 26 % |
|  | COx | 61 % | 79 % | 37 % |
| **B CDH Term** | Mxa | 3 % | 13 % | 8 % |
|  | COx | 0 % | 25 % | 31 % |
| **C Gastrointestinal atresia Preterm** | Mxa | 39 % | 38 % | 41 % |
|  | COx | 21 % | 44 % | 25 % |
| **D Other major Preterm** | Mxa | 59 % | 60 % | 61 % |
|  | COx | 83 % | 48 % | 41 % |
| **E CDH Term** | Mxa | 79 % | 78 % | 80 % |
|  | COx | 70 % | 60 % | 52 % |

The table show the mean percentage of time with impaired cerebral autoregulation assessed with mean flow index (Mxa) and cerebral oxygenation index (COx) in five infants. CDH: Congenital diaphragmatic hernia. P1: Anesthesia, before start surgery, P2: Surgery, P3: Recovery

Table S6: Infants with incomplete data across the monitoring periods and the reasons for data incompleteness

|  |  |  |  |
| --- | --- | --- | --- |
| **Missing periods** | **Number** | **Reasons** | **Type of surgery** |
| P3 | 1 | Philips monitor data missing during recovery | gastrointestinal atresia |
| P3 | 1 | No postoperative monitoring (long duration surgery*) | gastrointestinal atresia |
| P3 | 1 | No arterial line during recovery | other major surgery |
| P3 | 1 | No postoperative monitoring (long duration surgery**) | gastrointestinal atresia |
| P2 and P3 | 1 | Probe removed (fixation) before start surgery | gastrointestinal atresia |
| P1 and P2 | 1 | Philips monitor data missing during anesthesia and surgery | abdominal wall defects |
| P1 and P2 | 1 | Philips monitor data missing during anesthesia and surgery | gastrointestinal atresia |
| P1= anesthesia, before start of surgery, P2 = surgery, P3 = recovery, *13 hours, **12 hours | | |  |
